# Antimicrobial Activities of *Saponaria cypria* Boiss. Root Extracts, and the Identification of Nine Saponins and Six Phenolic Compounds

**DOI:** 10.3390/molecules27185812

**Published:** 2022-09-08

**Authors:** Despina Charalambous, Michalis Christoforou, Elina N. Kitiri, Marios Andreou, Dora Partassides, Christoforos Papachrysostomou, Myriam Frantzi, George A. Karikas, Maria Pantelidou

**Affiliations:** 1 Frederick Research Center, Nicosia 1036, Cyprus; 2 Department of Pharmacy, School of Health Sciences, Frederick University, Nicosia 1036, Cyprus; 3 Cosmetics and Food Supplements Lab, State General Laboratory, Ministry of Health, Nicosia 2081, Cyprus; 4 Veterinary Drug Residues Lab, State General Laboratory, Ministry of Health, Nicosia 2081, Cyprus

**Keywords:** *Saponaria cypria*, cyprus, phenolics, saponins, antibacterial

## Abstract

The purpose of this study was to identify the chemical components in root extracts of *Saponaria cypria*, an endemic species of Cyprus. Subsequently, the synergistic bioactivity of its root extracts through different extraction procedures was also investigated for the first time. A total of nine saponins, along with six phenolic compounds, were identified and quantified using the UHPLC/Q-TOF-MS method. Additionally, *S. cypria* root extracts demonstrated antibacterial potential against *Escherichia coli*, *Staphylococcus aureus*, *Enterococcus faecalis* and *Salmonella enteritidis. S. aureus* presented the highest susceptibility among all bacteria tested. These findings provide the first phytochemical data regarding the saponin, phenolic content and antimicrobial activity of *S. cypria* extracts, indicating that the Cyprus *saponaria* species is a rich natural source for bioactive compounds with a potentially wider bioactivity spectrum.

## 1. Introduction

*Saponaria* plants, also known as soapworts, belong to the family Caryophyllaceae. Their genus name is derived from the Latin word “*sapo*” which means soap, since the roots of some species are rich in active molecules called saponins [1]. Saponins are glycosylated molecules of an amphiphilic nature which form stable, soap-like foams in aqueous solutions [1,2]. They are composed of two main parts: a water-soluble glycosidic chain and a liposoluble structure. The non-sugar and sugar components are called aglycone and glycone portions, respectively. The aglycone portion is composed of a triterpenoid or a steroid backbone. The sugar moiety is linked to the aglycone through an ester or ether glycosidic linkage at one or more glycosylation sites [1,2].

In the past, soapwort extracts were used as household detergents and cosmetics, mainly due to the emulsifying, cleansing and foaming properties of its saponin components. Today, one of the major applications of the common species *Saponaria officinalis* L., is its use as a natural emulsifier in the production of halva, a popular confectionery. Besides food and cosmetics, the saponin-rich extracts demonstrate strong biological activity and may potentially be used as alternative medications for disorders such as heart disease, chronic inflammatory disease and cancer [2,3]. Saponins isolated from the roots of *S. officinalis*, have been previously characterized in terms of their chemical composition [4,5,6,7] and antibacterial activity [8,9,10]. Moreover, extracts from *S. officinalis* aerial parts have been reported to possess antioxidant properties, due to their rich content of phenolic compounds [8,11]. Besides their antioxidant activity, polyphenols found in many plant species, are also known for their anti-inflammatory [12,13], anti-diabetic [14], hypocholesterolemic [15] and antibacterial properties [16].

Although extracts from *S. officinalis* have been studied extensively, the endemic *Saponaria* species of Cyprus, known as *Saponaria cypria* Boiss., has not been previously investigated in terms of its saponin and phenolic content or its biological properties. *S. cypria* is an erect or spreading perennial plant, 10–30 cm high, with a thick branched woody rootstock and basal leaves loosely clustered, glabrous or thinly ciliate at the base of the petiole, spathulate, obovate or oblanceolate (2.5–5 cm long, 0.5–1.5 cm wide) [17]. Its flowers are actinomorphic, solitary or in cymes, having a pink color, and they are deeply emarginated. The plant blooms from July to September, and grows on rocky areas with forest openings, screes, road banks, and by streams at an altitude of 1100–1950 m. Its distribution is confined to the Troodos Forest in areas such as Chionistra, Prodromos, Trooditissa and Xerokolymbos [18]. 

Up to now, the chemical profile, as well as the biological properties, of *S. cypria* have not been evaluated. Thus, the aim of this work was to study the chemical content in terms of specific saponin and phenolic compounds of the root extracts of *S. cypria*. Further to the identification of saponin and phenolic components, the antibacterial activity of the root extracts was tested against gram-positive and gram-negative bacteria. 

## 2. Results

### 2.1. Determination of Total Saponin Content

The total saponin content (TSC) of *S. cypria* root extracts was determined using three different solvents (methanol, ethanol and acetone), based on previously described methods [19]. A standard curve of oleanolic acid was constructed and the results were expressed as mg oleanolic acid equivalents per gram of dry crude extract (mg OAE/g crude extract). According to the results, as shown in Table 1, acetone gave the highest TSC yield (169.000 mg OAE/g crude extract), significantly higher than the ethanol and methanol yields (106.210 and 64.331 mg OAE/g crude extract respectively, *p* < 0.01). 

### 2.2. Identification and Quantification of Saponins in S. cypria 

**Figure 1:**
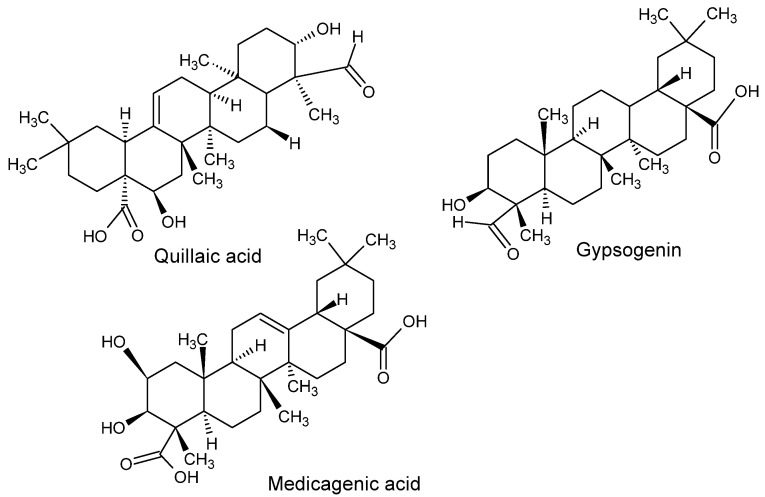
Structures of Quillaic acid, Gypsogenin and Medicagenic acid.

The saponin compounds identified in acetone *S. cypria* root extracts, using ultra-high performance liquid chromatography coupled to quadrupole time of flight mass spectrometry (UHPLC-QTOF-MS), are presented in Table 2, and the total ion chromatogram is documented as supplementary material (Figure S1). The MS/MS fragmentation patterns and chromatograms of each compound are presented in Figure S2. According to the obtained results, a total of nine major saponins were identified, belonging to triterpene saponins. These are glycosylated derivatives of triterpene sapogenin, the aglycone moiety of each compound. The mass spectrometry analysis of the saponin compounds allowed the total identification of the compounds by direct comparison to previously published data on *S. officinalis* saponin fragmentation [4,5,7,20,21]. Saponarioside A (compound **5**) and other saponins derived from Quillaic acid (compounds **4**, **6**), Medicagenic acid (compounds **1**, **2**, **3**) and Gypsogenin (compounds **7**, **8**, **9**) were identified (Table 2). Structures of the backbone of these saponins are shown in Figure 1. 

Although the three saponins (compounds **1**, **2**, **3**), Table 2 have been previously reported [22], this is the first time that these compounds were found in a *Saponaria* species. In these cases, fragmentation patterns revealed the ions at *m*/*z* 501.3185 which is characteristic of Medicagenic acid as the aglycone moiety. The additional fragment ions at *m/z* 485.147 and 439.3183 were documented according to Peeters et al. [22].

Compounds **4** and **6** with retention times of 10.06 and 11.57 min and *m*/*z* of 1729.7330^2−^ and 1657.6978^2−^, respectively, were also identified. Compound **4** revealed fragment ions at *m*/*z* 955.4468, characteristic of Quillaic acid backbone with a loss of a pentose (*m*/*z* 132/150), three desoxyhexoses (*m*/*z* 146), one hexose (*m*/*z* 162/180) and one acetyl unit (*m*/*z* 42/60). The fragment ion observed at *m*/*z* 113.0231 was considered to be produced from hexoses. Compound **6** also revealed fragment ions at *m*/*z* 955.4468 and 113.0253 and an additional ion at *m*/*z* 485.3222 with a loss of a pentose (*m*/*z* 132/150), three desoxyhexoses (*m*/*z* 146), one hexose (*m*/*z* 162/180), and uronic acid (*m*/*z* 176) [23]. Based on the molecular weight and the fragmentation pattern of these two compounds, which were compared to the values of signature fragment ions of Quillaic acid octosaccharide and Quillaic acid heptasaccharide previously described in the literature [7], compounds **4** and **6** were proposed to be Quillaic acid octosaccharide and Quillaic acid heptasaccharide, respectively.

Compound **5** (retention time 10.75 min) was identified as Saponarioside A with an ion [M-H]^−^ of 1699.7172^2−^ and fragment ions at *m*/*z* 1681.7118, 1567.6679, 955.4555 and 469.1593 (Table 2), as previously reported in literature [7].

Compounds **7** and **8** with the molecular formula of C_73_H_120_O_43_ were detected at retention times 14.00 and 14.43 min, respectively (Table 2). These compounds demonstrated the same fragmentation pattern with produced ions at *m*/*z* 1551.6802, 939.4517 and 469.3272, which are characteristic of the Gypsogenin backbone, as documented in the literature [7]. 

Finally, compound **9**, another Gypsogenin derivative, was identified at retention time 15.82 min and *m*/*z* of 1447.6217^2−^ (C_64_H_104_O_36_). This was regarded as Gypsogenin hexasaccharide, based on the MS/MS data, which provided fragment ions at *m/z* 939.4448, 469.3299 and 113.0223. According to the literature this compound has been previously identified in *S. officinalis* extracts [7].

Quantification analysis revealed that the major saponin components of the extract are the Medicagenic acid derived saponin (compound **3**, *m*/*z* 1293.5673) and the Gypsogenin derivative (compound **9**) at 2.588 % and 2.447 %, respectively (Table 2).

### 2.3. Determination of Total Phenolic Content 

The total phenolic content (TPC) of methanol, ethanol and acetone root extracts of *S. cypria* was detected by using the Folin-Ciocalteu method [24]. A standard curve of gallic acid was constructed and the results were expressed as mg gallic acid equivalents per gram of crude extract (mg GAE/g). According to the data presented in Table 3, the *S. cypria* acetone extract demonstrated the highest TPC result (21.016 mg GAE/g crude extract), a yield significantly higher than the methanol and ethanol extracts (*p* < 0.01).

### 2.4. Identification and Quantification of Phenolic Compounds in S. cypria

The phenolic compounds identified in the acetone *S. cypria* root extract, using UHPLC-QTOF-MS/MS, are presented in Table 4 and the total ion chromatogram is documented as supplementary material (Figure S3). The MS/MS fragmentation pattern and chromatograms of all identified compounds are also provided as supplementary material (Figure S4). Six phenolic compounds were identified, including Rutin, Quercetin glucosides, Syringic acid and 4,5-di-O-Caffeoylquinic acid. The structural identification of these compounds was based on a comparison of their MS/MS data with those reported in the literature [25,26]. 

Compound **1** (retention time, 4.52 min), which demonstrated an *m*/*z* of 359.0986, was assigned as Syringic acid O-hexoside based on three main fragment ions at *m*/*z* 197.0455, 153.0555 and 149.0237 [25]. 

Compound **3** generated an [M-H]^−^ ion at *m*/*z* 609.1456 (C_27_H_30_O_16_). In the secondary mass spectrum, *m*/*z* 300.0277, 178.9991 and 151.0035 were the characteristic ions produced by fragmentation. By comparison to the literature [26], compound **3** was identified as Rutin. 

Compound **2**, with a generated formula C_33_H_40_O_20_, retention time of 6.07 min and *m*/*z* 755.2028, gave no fragmentation pattern. By comparing these results to previously reported data [25], this compound was identified as Quercetin 3-*O*-(2,6-di-*O*-rhamnosyl-glucoside). Compound **4**, with a generated formula C_21_H_20_O_11_, retention time of 7.50 min and *m*/*z* 447.0922, gave three characteristic product ions (Table 4). A comparison of these data to the literature [25] suggests that this compound is Quercetin 3-*O*-rhamnoside (quercitrin). In a similar manner, the molecular formula and fragmentation pattern of compound **6** indicated that this compound seemed to be Quercetin 3-*O*-galactoside [25]. 

Finally, compound **5** (retention time, 7.67 min), which generated an ion [M-H]^−^ of 515.1192, and produced fragment ions at *m*/*z* 353.0868 and 179.0343 (Table 4), was identified as Caffeoylquinic acid [25]. Quantification analysis revealed that among the phenolic compounds identified, Caffeoylquinic acid (compound **5**) was the major constituent detected at 1.855 % (Table 4).

### 2.5. Antimicrobial Activity of Extracts

The minimum inhibitory concentration (MIC) and minimum bactericidal concentration (MBC) of methanol, ethanol and acetone root extracts of *S. cypria* were evaluated against gram-negative (*E. coli, S. enteritidis*) and gram-positive bacteria (*S. aureus, E. faecalis*). According to the results shown in Table 5, all *S. cypria* extracts demonstrated bacterial inhibition, with MIC values ranging from 0.195–1.563 mg/mL for *S. aureus* and 0.391–3.125 mg/mL for *E. faecalis*, while the inhibition activities against *E. coli* and *S. enteritidis* were the weakest (3.125 mg/mL). *S. cypria* acetone extract exhibited the highest bacterial inhibition against *S. aureus* (MIC, 0.195 mg/mL) and *E. faecalis* (MIC, 0.391 mg/mL). 

The antimicrobial efficacy was also studied by determining MBC, which is defined as the lowest concentration of the extract that is bactericidal. Therefore, the lower the MBC value, the less extract is needed to kill the bacteria. *S. cypria* exhibited low MBC values (more bactericidal) ranging from 0.195–1.563 mg/mL for *S. aureus* and 0.391–3.125 mg/mL for *E. faecalis*, whereas the bactericidal effects on *E. coli* and *S. enteritidis* were weaker (6.250 mg/mL for *E. coli* and values ranging from 6.250 to 12.500 mg/mL for *S. enteritidis*)*. S. cypria* acetone extract exhibited the lowest MBC value against *S. aureus* (0.195 mg/mL). 

## 3. Discussion

The present study is the first attempt that documents data regarding the saponin and phenolic chemical profiles of *S. cypria* root extracts. Further to the molecules detected, our results also provide valuable evidence for antibacterial activity.

Although the endemic species was the main focus of this study, it is important to note that two other species, namely *Saponaria mesogitana* Boiss. and *Saponaria orientalis* L., are also encountered on the island. *S. cypria* can be identified and distinguished from *S. mesogitana* and *S. orientalis* based on morphological characteristics [17,27]. The main difference between *S. cypria* and *S. orientalis* is that the endemic taxon is perennial with woody rootstock, while *S. orientalis* is annual [17]. Apart from that, there is an obvious difference regarding the diameter of the flowers since the endemic taxon has much larger flowers; similarly, there is a distinct difference concerning the size of the calyx [17]. *S. mesogitana* is an annual plant and has two short coronal scales, a characteristic that is not found in *S. orientalis* [27]. 

Regarding the extraction procedure implemented in the current study, three different solvents were used, namely methanol, ethanol and acetone. Thus, a comparison of the total saponin yield extracted with each solvent revealed that acetone exhibited the highest saponin yield, a finding which was in agreement with previously reported data regarding the saponin content of safed musli extracts [28]. According to Barve *et al.*, this may be attributed to the polar and non-polar properties of acetone which may justify a higher extraction yield of saponins compared to ethanol or methanol [28]. 

Although there is no reported data regarding the saponin content of *S. cypria* species, previously published results documented the saponin content of the root of *S. officinalis* with a value of 82.4 mg/g crude root extract [7]. Our results may suggest that *S. cypria* root extract is richer in saponins than *S. officinalis*, however different extraction and quantification methods were implemented by Budan et al. compared to our study, which may have contributed to the different TSC values observed. Moreover, apart from dealing with different *Saponaria* species, the total saponin yield may also be affected by environmental factors, such as the following: micro-climate, temperature cultivation period, geographical location, and growth conditions [29,30]. According to the literature, *Saponaria* species are considered a good source for saponins with a content close to 10%. Although most reported species seem to have a lower content, for instance, Soybean (0.22–0.47%), Chickpea (0.23%), Alfalfa (0.14–1.71%) or Quinoa (0.14–2.3%), there are several other species which seem to have considerable amounts, such as Quillaja bark (10%) and Yucca (10%) [30,31,32]. Interestingly, Licorice root and the American Ginseng have been reported to be a rich source of saponins (22.2–32.3%) [30,31,32].

In relation to our results, nine major saponins were identified in the *S. cypria* root extracts. These included Saponarioside A and saponins derived from Quillaic acid, Medicagenic acid and Gypsogenin. Among the saponin molecules identified in the current study, relatively higher quantities of Medicagenic acid and Gypsogenin conjugates (compound **3** and **9** respectively) were observed. Saponins derived from Medicagenic acid have not been previously reported in other *saponaria* species. However, all structurally known saponins have been previously identified in various plant species and most of them have been studied for their biological roles. For instance, Quillaic acid saponins, also known as *Quillaja* saponins, have been reported to possess anti-inflammatory, antibacterial and antiviral activity [33]. Furthermore, Quillaic acid and Gypsogenin isolated from *S. officinalis* roots, have been reported to have antiproliferative properties by inhibiting the growth of tumorigenic human breast cancer and prostate cancer cells [3]. Medicagenic acid detected in other plant species, demonstrated antibacterial, as well as antifungal properties [34,35]. Interestingly, among the saponins identified in our study, the components derived from Medicagenic acid had the highest quantity in the extract of *S. cypria*.

Concerning the antibacterial properties, the present study demonstrated the bacterial inhibition of *S. cypria* root extracts against all four strains tested, namely *E. coli*, *S. aureus*, *E. faecalis* and *S. enteritidis.* Although this is the first time *S. cypria* species was tested for its antibacterial potential, the antimicrobial properties of other *Saponaria* species have been reported in the literature. More specifically, methanol extracts of *S. officinalis* were reported to demonstrate antibacterial activity against *S. aureus* and *E. faecalis* [8,9,10]. Similar to our study, *Saponaria prostrata* Willd. extracts demonstrated the highest antibacterial activity against *S. aureus* among various gram-positive and gram-negative bacteria tested and, unlike *S.* cypria, it did not express antimicrobial activity against *E. coli* [36]. Other studies reported that the *Sapindus saponaria* L. hydromethanolic extract is effective against various fungal and bacterial strains, with best activity against *Bacillus cereus* and *S. aureus* [37], while the ethanolic extract of *Sapindus saponaria* Vahl also seemed effective against all tested bacterial pathogens including *S. aureus* [38].

A great deal of attention has also been given to natural antioxidants and their health benefits during the past few years. Knowing that polyphenols are the most abundant antioxidant molecules in nature, this study also aimed at investigating the presence of phenolic compounds in *S. cypria* root extracts. The results confirmed that S. *cypria* root is also a source of phenolic compounds. A total of six phenolic compounds were identified in the *S. cypria* plant. The extract presented high amounts of Caffeoylquinic acid, a plant metabolite which has been described as an antibacterial agent against gram-positive *Bacillus cereus* and *S. aureus* in the past [39], as well as a free radical scavenger in a study using Coffee silver skin extracts [40]. Other phenolic compounds detected at lower concentrations included Quercetin glucosides, Rutin and Syringic acid. Quercetin, a well-known flavonoid is found in many plants. In fact, Quercetin O-glycoside derivatives are well known for their antioxidant properties [41]. Additionally, the 3-O-rutinoside derivative of Quercetin, named Rutin, is found in several species of the Caryophyllaceae and it has been reported to have a wide range of biological properties [42,43,44]. Syringic acid, another phenolic compound identified in this study, has also been reported to demonstrate a wide range of health-related properties, such as prevention of oxidative stress [45,46] and antimicrobial activities against several gram-positive and gram-negative bacteria [47]. Although the extracts of *S. officinalis* have been previously reported to contain phenolic compounds and, particularly, flavonoids, this is the first study to provide data on the phenolic content of *S. cypria* species and to demonstrate that the root of the plant is a good source of antioxidant and antimicrobial agents. Apart from the obvious synergistic effects, the presence of phenolic Caffeoylquinic acid and saponin Medicagenic acid could, at this stage, explain the significant antimicrobial activities of *S. cypria.*

In conclusion, the above results contribute towards the phytochemical and pharmacological knowledge regarding *S. cypria*, as well as its promising synergistic actions that may in the future be of great use as alternative medicine and nutritional supplements. Further studies, which are currently underway, will help elucidate the total content of bioactive compounds of *S. cypria*. Furthermore, tests using in vitro biological assays, e.g., cell lines, or *in vivo* assessments, are required to help determine the antioxidant activity of isolated phenolics. Overall, this study provides valuable data for the exploitation of *S. cypria* by the pharmaceutical and cosmetic industries.

## 4. Material and Methods

### 4.1. Plant Material

Sampling was carried out with the coordinates of the central point of the surface being as follows: x: 485,399; y: 3,866,330; z: 1358 (in UTM system 36S). *S. cypria* plants were identified and distinguished from other *Saponaria* species based on morphological characteristics, as previously described [17,27]. Roots were collected from five randomly selected mature *S. cypria* plants (total dry root mass = 500 g), and cultivated at the nurseries of the Department of Forests in Troodos, Cyprus. Cultivated plants came from seeds germinated at the Nature Conservation Unit at Frederick Research Center. Seeds of *S. cypria* came from two seed banks in Cyprus (the Agricultural Research Institute Gene-bank (Nicosia, Cyprus) and the Nature Conservation Unit Seedbank (Nicosia, Cyprus). 

### 4.2. Preparation of Extracts

*S. cypria* roots were washed, air-dried at room temperature for 3–4 days and crushed into fine powder. Three different solvents were used: 100% methanol (Merck, Gillingham, UK), 100% ethanol (Merck, Gillingham, UK), and 100% acetone (Merck, Gillingham, UK). Powder (10 gr each time) was added to 150 mL solvent and macerated continuously at room temperature for 24 h. Thereafter, the extracts were centrifuged at 4 °C, 4000 rpm for 10 min and filtered. The solvent in each extract was fully evaporated using a rotary evaporator (Stuart RE300, Keison, Chelmsford, UK) at 60 °C under vacuum of <1 mmHg. The remaining solids were redissolved in methanol. The crude extracts were stored at 4°C until further analysis.

### 4.3. Total Saponin Content

The total saponin content (TSC) of *S. cypria* root extracts was measured, as previously described [19]. In a glass tube, 250 μL of each extract was added along with 1 mL of reagent mix containing glacial acetic acid (Merck, Gillingham, UK) and sulfuric acid (1:1, *v*/*v*, Sigma Aldrich, Hamburg, Germany). The contents of the tube were vortexed vigorously and heated at 60 °C for 30 min during which a purple color developed. Following incubation, the tubes were rapidly cooled to room temperature in an iced water bath. The absorbance of all samples was measured at 527 nm (UV-1280, Shimadzu Europa GmbH, Duisburg, Germany). A standard oleanolic acid (Sigma Aldrich, Hamburg, Germany) curve (0.1–1 mg/mL) was constructed. The TSC of all extracts was expressed as mg of oleanolic acid equivalents per gram of crude extract (mg OAE/g crude extract) using the linear regression equation of the oleanolic acid standard curve. All experiments were performed in triplicate and the results were expressed as the mean value ± standard deviation (SD).

### 4.4. UHPLC-QTOF-MS Analysis 

The identification of the saponin components and the phenolic compounds was performed by UHPLC-QTOF-MS, (Agilent Technologies, Santa Clara, CA, USA). The gradient elution steps were: 98% A, water (Water for LCMS, Carlo Erba, Italy), (0–0.5 min), 98% to 2% A (19 min), 2% A (24 min), 2% to 0% A (26 min), 100% B, acetonitrile (Carlo Erba, Italy) (29 min), 100% to 2% B (30 min) and 98% A (35 min), modifier 0.1% formic acid, (Carlo Erba, Italy) in both, the injection volume was 10 μL and the flow rate was 0.3 mL/min. The liquid chromatography was performed with an Agilent 1290 Infinity LC system (Agilent Technologies, Santa Clara, CA, USA) and the separation of the saponins was achieved using a Waters Sunfire column, 150 mm × 2.1 mm, 3.5 μm, at 40 °C, (Waters Corporation, Milford, MA, USA). The MS experiments were performed on an Agilent 6550 iFunnel high resolution quadrupole time of flight mass spectrometer operating in the negative mode using default settings, (Agilent Technologies, Santa Clara, CA, USA). All chromatographic data were acquired in MS and AutoMS/MS mode using collision energies at 10, 20, 40 and 60 volts. The MS/MS data were processed with the MassHunter Workstation Software known as Qualitative Analysis Version B.06.00. The molecular formula assignment was carried out for each identified compound by comparing the experimental *m*/*z* to theoretical values, allowing a mass error of less than 5 ppm. The mass error of all fragment ions was also less than 5 ppm. The molecular weight values and the fragmentation pattern of the compounds were compared to previously reported values of signature ion fragments of known saponins [7,22] and phenolics [25,26]. The structures of saponin backbones were prepared using the ChemSketch program (ACD/Labs Toronto, Canada). Relative quantification was based on calculated peak areas of the nine saponins using the linear regression response curve of reference Quillaic acid (Sigma Aldrich, Germany). Similarly, the linear regression response curve of reference Quercetin (Sigma Aldrich, Germany) was used for the quantification of the six phenolic compounds. The standard concentration range used for quantification was 5, 10, 50, 100, 200, 400, 600 and 800 ng/injection for Quillaic acid and 5, 10, 50, 100, 200 and 400 ng/injection for Quercetin. The data was presented as the mean % (g of compound per 100 g of crude extract) ± the estimated standard deviation (SD) of three independent experiments.

### 4.5. Total Phenolic Content

The total phenolic content (TPC) of *S. cypria* root extracts was determined using the Folin-Ciocalteu method, as previously described [24]. A standard gallic acid (Sigma Aldrich, Hamburg, Germany) curve was constructed by preparing dilutions of 0.05–0.4 mg/mL in methanol (Merck, Gillingham, UK). In a glass tube, 100 μL of each of these dilutions were mixed with 500 μL water and then 100 μL of Folin-Ciocalteu reagent (Sigma Aldrich, Hamburg, Germany). Each reaction mixture was allowed to stand for 6 min, followed by the addition of 1 mL of 7% sodium carbonate (Sigma Aldrich, Hamburg, Germany) and then 500 μL of distilled water. The absorbance was recorded after 90 min spectrophotometrically at 760 nm (UV-1280, Shimadzu Europa GmbH, Duisburg, Germany). The same procedure was repeated with *S. cypria* extracts. The TPC of all samples was expressed as mg of gallic acid equivalents per gram of crude extract (mg GAE/g crude extract) using the linear regression equation of the gallic acid standard curve. All experiments were performed in triplicate and the results were expressed as the mean value ± standard deviation (SD).

### 4.6. Antibacterial Activity

#### 4.6.1. Minimum Inhibitory Concentration 

The broth microdilution method was used for the determination of MIC of the *S. cypria* root extracts. *Saponaria* extracts as a 50 mg/mL starting solution were subjected to 2-fold serial dilutions. Specifically, 200 μL of each extract (50 mg/mL) were added as a starting solution and 2-fold serial dilutions with Tryptic Soy broth (TSB, Liofilchem, Italy) were prepared. Isolated cultures of *E. coli* (NCTC 9001, Sigma Aldrich, Hamburg, Germany), *S. aureus* (NCTC 6571, Sigma Aldrich, Germany), *E. faecalis* (NCTC775, Sigma Aldrich, Hamburg, Germany) and *S. enteritidis* (WDCM 00030, Sigma Aldrich, Hamburg, Germany) were prepared in TSB at a concentration of approximately 1 × 10^6^ cfu/mL. One-hundred microliters (100 µL) of each bacterial inoculum were added in each well, containing either extract or controls. Blank samples of each extract (containing no bacteria) were subjected to 2-fold serial dilution with TSB (blank control). Control samples included bacteria (100 μL), but no extracts were used as growth controls. A sterility control was used with TSB, no bacteria and no extract. A well with bacteria and Ampicillin (0.516 mg/mL, Sigma Aldirch, Hamburg, Germany) or Gentamycin (0.064 mg/mL, Molekula, Darlington, UK) were used as positive controls. The MIC of each sample was detected after 18 h of incubation at 37 °C, followed by the addition of 30 µL (0.2 mg/mL) p-iodonitrotetrazolium chloride (INT, Sigma Aldrich, Gillingham, UK) and incubation at 37 °C for 30 min. The absorbance at 492 nm was measured with a microplate reader (Sunrise, Tecan Trading Ltd., Mannedorf, Switzerland). The MIC of each extract was defined as the minimum sample concentration that prevented the color change of the medium, thus exhibiting complete inhibition of bacterial growth as compared with that of the blank control.

#### 4.6.2. Minimum Bactericidal Concentration 

The MBC of *S. cypria* extracts was determined by sub-culturing 2 μL aliquots of the preparations from the MIC assay in 100 μL TSB and incubating for 24 h at 37 °C. The MBC was defined as the lowest concentration of each sample not exhibiting color change, after addition of INT, as described above.

### 4.7. Statistical Analysis

All experiments were performed in triplicates and the results were expressed as the mean value ± the estimated SD. Significance between the means was determined by student’s *t* test (*p* < 0.01).

## Figures and Tables

**Table 1:** Total Saponin Content of methanol, ethanol and acetone root extracts of *S. cypria*.

Solvent Type	TSC (mg OAE ^1^/g Crude Extract) ± SD
Methanol	64.331 ^c^ ± 2.040
Ethanol	106.210 ^b^ ± 4.167
Acetone	169.000 ^a^ ± 7.155

^1^ mg OAE/g crude extract: mg oleanolic acid equivalents per gram of dry crude extract; TSC: Total Saponin Content; SD: Standard deviation. ^a–c^ Values having different letters differ significantly (*p* < 0.01).

**Table 2:** UHPLC-QTOF-MS mass spectra data in negative ion mode of the major saponin compounds identified in *S. cypria* root extracts.

Compound Number	RT	Molecular Formula	Observed Ion *m/z* [M-H]^−^	MS/MSProduct Ions	Compound Name	Saponin Content WT % ± SD	References
**1**	8.86	C_66_H_104_O_35_	1455.6156	1275.5501, 1231.5589, 1149.5248, 969.4606, 501.3174,439.3152, 485.1478, 323.0953, 179.0549, 113.0231	MA	0.005 ± 0.001	[22]
**2**	9.23	C_54_H_86_O_26_	1149.5256	969.4637, 501.3194, 483.3075, 439.3187, 485.1478, 341.1074, 323.0969, 113.0241	MA	0.113 ± 0.082	[22]
**3**	9.57	C_60_H_94_O_30_	1293.5673	1113.5009, 969.4701, 501.3185, 439.3183, 485.1477, 341.1099, 323.0953, 113.0262	MA	2.588 ± 0.091	[22]
**4**	10.06	C_78_H_122_O_42_	1729.7330^2−^	955.4468, 113.0226	QA octosaccharide	0.005 ± 0.001	[7]
**5**	10.75	C_77_H_120_O_41_	1699.7172^2−^	1681.7118, 1567.6679, 955.4555, 469.1593	Saponarioside A	0.034 ± 0.012	[7,21]
**6**	11.57	C_71_H_118_O_43_	1657.6978^2−^	955.4449, 485.3222, 113.0253	QA heptasaccharide	1.762 ± 0.065	[7]
**7** *	14.00	C_73_H_120_O_43_	1683.7169^2−^	1551.6802, 939.4517, 469.3272	G octasaccharide	1.855 ± 0.081	[7]
**8** *	14.43	C_73_H_120_O_43_	1683.7114^2−^	1551.6691, 939.4502, 469.3272	G octasaccharide	1.527 ± 0.076	[7]
**9**	15.82	C_64_H_104_O_36_	1447.6217^2−^	939.4448, 469.3299, 113.0223	G hexasaccharide	2.447 ± 0.069	[7]

RT: retention time; *m*/*z* [M-H]^−^: value of deprotonated molecule; ^2−^: *m*/2*z* ion detected; WT %: weight percentage (g component per 100 g dry root); SD: Standard deviation; MA: Medicagenic acid; QA: Quillaic acid; G: Gypsogenin; *compounds having isomers with identical mass spectral data but different retention time.

**Table 3:** Total Phenolic Content of methanol, ethanol and acetone *S. cypria* root extracts.

Solvent Type	TPC (mg GAE ^1^/g Crude Extract) ± SD
Methanol	13.623 ^b^ ± 0.183
Ethanol	12.156 ^b^ ± 0.262
Acetone	21.016 ^a^ ± 0.357

Results were expressed as the mean values of three independent experiments. ^1^ mg GAE/g crude extract: mg gallic acid equivalents per gram of crude extract; TPC: Total Phenolic Content; SD: Standard deviation. ^a–b^ Values having different letters differ significantly (*p* < 0.01).

**Table 4:** UHPLC-QTOF-MS mass spectra data in negative ion mode of phenolic compounds identified in *S. cypria* root extracts.

Compound Number	RT	Molecular Formula	Observed Ion *m*/*z* [M-H]^−^	MS/MS Productions	Compound Name	Phenolic Compounds Content WT % ± SD	Reference
**1**	4.52	C_15_H_20_O_10_	359.0986	197.0455, 153.0555, 149.0237	Syringic acid *O*-hexoside	0.298 ± 0.108	[25]
**2**	6.07	C_33_H_40_O_20_	755.2040	755.2028	Quercetin 3-*O*-(2,6-di-*O*-rhamnosyl-glucoside)	0.244 ± 0.112	[25]
**3**	6.55	C_27_H_30_O_16_	609.1459	300.0277, 178.9991, 151.0035	Rutin	0.231 ± 0.084	[26]
**4**	7.50	C_21_H_20_O_11_	447.0922	301.0338, 300.0265, 271.0245, 255.0300, 151.0035	Quercetin 3-*O*-rhamnoside (quercitrin)	0.712 ± 0.072	[25]
**5**	7.67	C_25_H_24_O_12_	515.1192	353.0868, 179.0343	4,5-di-*O*-Caffeoylquinic acid	1.855 ± 0.126	[25]
**6**	7.87	C_21_H_20_O_12_	463.0885	301.0354, 300.0267, 273.0405, 151.0036,	Quercetin 3-*O*-galactoside	0.095 ± 0.01	[25]

RT: retention time; *m*/*z* [M-H]^−^: value of deprotonated molecule; WT %: weight percentage (g component per 100 g dry root); SD: Standard deviation.

**Table 5:** Minimum inhibitory concentration (MIC) and minimum bactericidal concentration (MBC) for *S. cypria* methanol, ethanol and acetone root extracts against *E. coli, S. aureus*, *E. faecalis* and *S. enteritidis*.

	*E. coli*			*S. aureus*			*E. faecalis*			*S. enteritidis*			Amp ^1^ (Control)	Gen ^1^ (Control)
	MEOH	ETOH	ACE	MEOH	ETOH	ACE	MEOH	ETOH	ACE	MEOH	ETOH	ACE	-	-
MIC ^2^ (mg/mL)	3.125	3.125	3.125	1.563	0.391	0.195	3.125	1.563	0.391	3.125	3.125	3.125	0.004	0.004
MBC ^3^ (mg/mL)	6.250	6.250	6.250	1.563	0.391	0.195	3.125	1.563	0.391	12.500	6.250	6.250	0.004	0.008

^1^ Ampicillin and gentamycin were used as control antimicrobial agents against *E. coli*/*S. enteritidis* and *S. aureus/E. faecalis,* respectively. ^2^ The lower the MIC value, the less extract is needed for inhibiting the growth of the bacteria. ^3^ MBC is the lowest concentration of the extract that is bactericidal. The lower the MBC value, the less extract is needed to kill the bacteria. Amp: Ampicillin; Gen: Gentamycin; MIC: Minimum Inhibitory Concentration; MBC: Minimum Bactericidal Concentration; MEOH: methanol solvent; ETOH: ethanol solvent; ACE: acetone solvent.

## Data Availability

Data reported in this study are contained within the article. The underlying raw data are available on request from the corresponding author.

## Supplementary Materials

The following supporting information can be downloaded at: https://www.mdpi.com/article/10.3390/molecules27185812/s1, Figure S1: UHPLC-QTOF-MS Extracted Ion Chromatogram of saponins of *S. cypria* root extract. Only peaks that represent saponins are indicated with numbers 1–9. Other peaks did not provide any evidence that they are saponins; Figure S2: MS/MS spectra data of saponins with precursor and product ions in negative mode; Figure S3: UHPLC-QTOF-MS Extracted Ion Chromatogram of phenolic compounds of *S. cypria* root extract. Only peaks that represent phenolic compounds are indicated with numbers 1–6. Other peaks did not provide any evidence that they are phenolic compounds; Figure S4: MS/MS spectra data of phenolic compounds with precursor and product ions in negative mode.

## Sample Availability

Samples of the *Saponaria cypria* extracts are available from the authors.
