# Supplementary material for: Antimicrobial Activities of Saponaria cypria Boiss. Root Extracts, and the Identification of Nine Saponins and Six Phenolic Compounds
Source: Molecules. 2022 Sep 8;27(18):5812. doi: 10.3390/molecules27185812 (PMC9505039; doi:10.3390/molecules27185812)
Supplement: Supplementary file 1 [file molecules-27-05812-s001.zip › molecules-1802425-supplementary.pdf]

Supplementary Materials

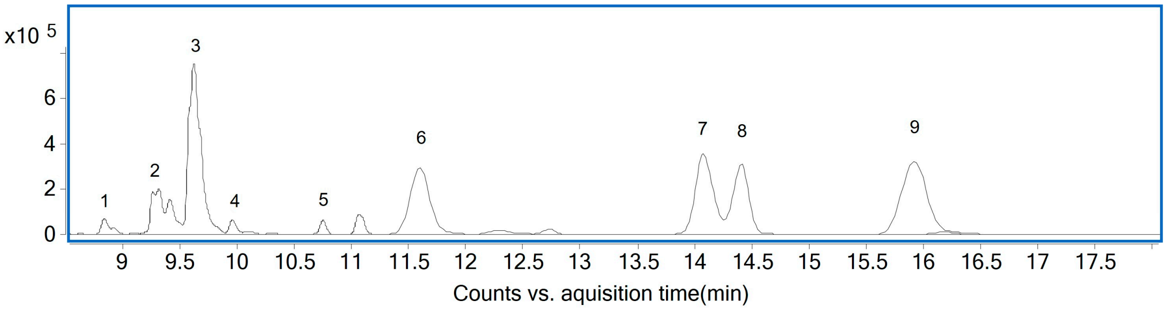

Figure S1. UHPLC-QTOF-MS Extracted Ion Chromatogram of saponins of *S. cypria* root extract. Only peaks that represent saponins are indicated with numbers 1-9. Other peaks did not provide any evidence that they are saponins.

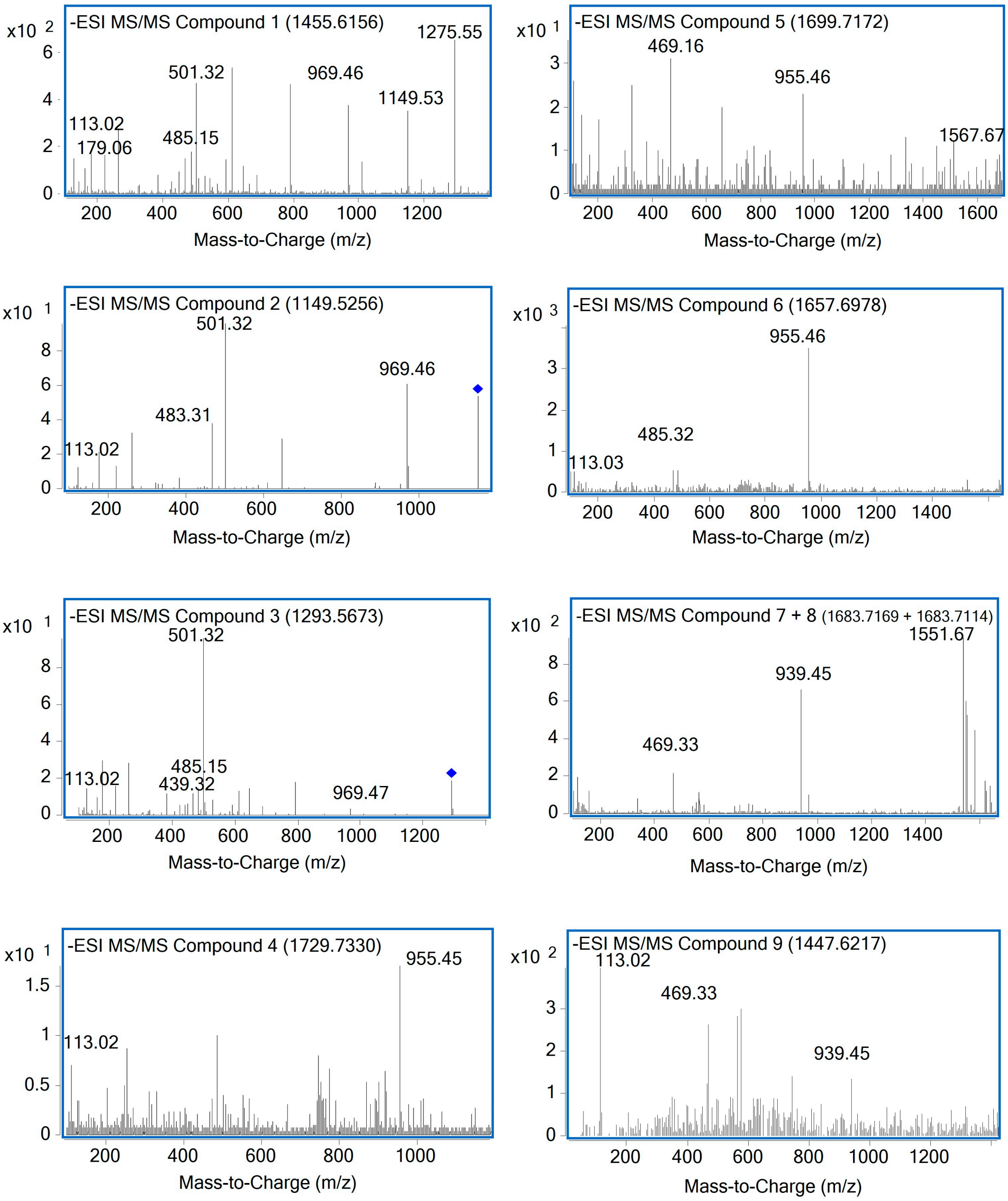

Figure S2. MS/MS spectra data of saponins with precursor and product ions in negative mode.

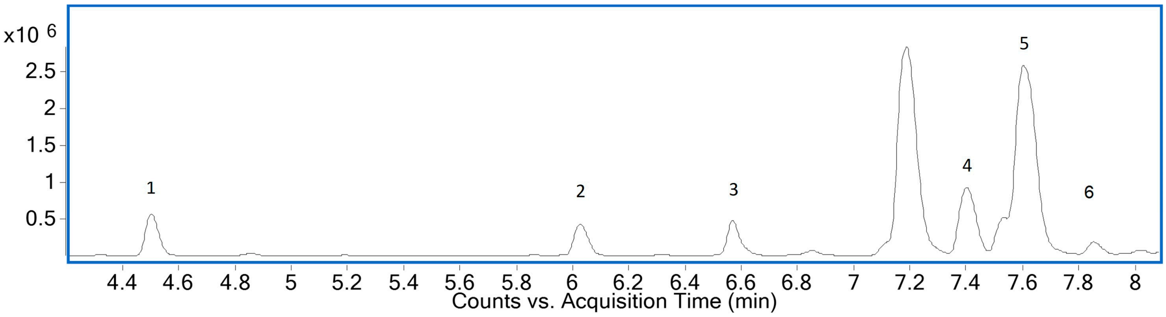

Figure S3. UHPLC-QTOF-MS Extracted Ion Chromatogram of phenolic compounds of *S. cypria* root extract. Only peaks that represent phenolic compounds are indicated with numbers 1-6. Other peaks did not provide any evidence that they are phenolic compounds.

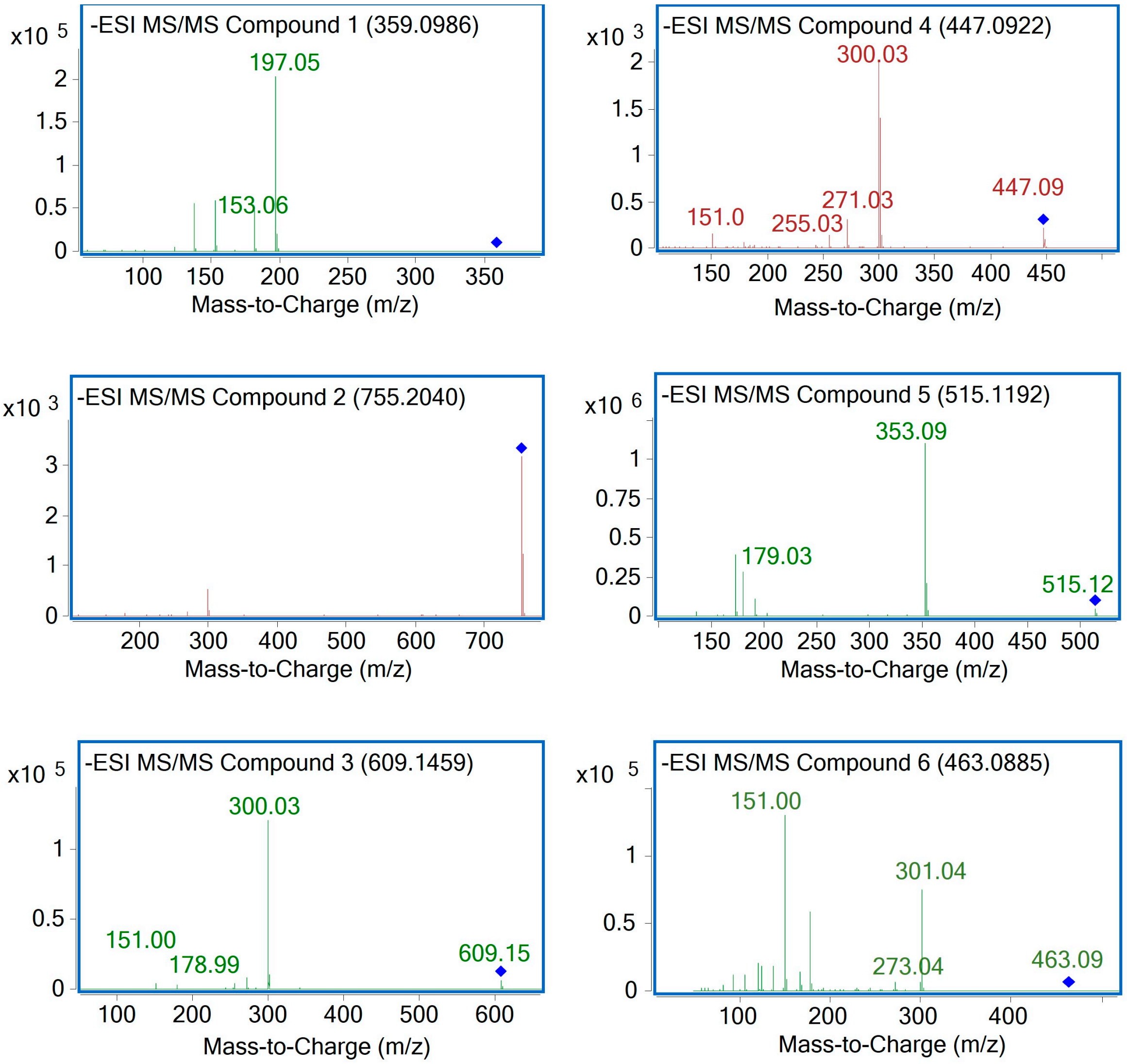

Figure S4. MS/MS spectra data of phenolic compounds with precursor and product ions in negative mode.
